# Design of a Zinc-Finger Hydrolase with a Synthetic αββ Protein

**DOI:** 10.1371/journal.pone.0096234

**Published:** 2014-05-09

**Authors:** Kinshuk Raj Srivastava, Susheel Durani

**Affiliations:** Department of Chemistry, Indian Institute of Technology Bombay, Mumbai, India; Oak Ridge National Laboratory, United States of America

## Abstract

Recent advances in protein design have opened avenues for the creation of artificial enzymes needed for biotechnological and pharmaceutical applications. However, designing efficient enzymes remains an unrealized ambition, as the design must incorporate a catalytic apparatus specific for the desired reaction. Here we present a de novo design approach to evolve a minimal carbonic anhydrase mimic. We followed a step-by-step design of first folding the main chain followed by sequence variation for substrate binding and catalysis. To optimize the fold, we designed an αββ protein based on a Zn-finger. We then inverse-designed the sequences to provide stability to the fold along with flexibility of linker regions to optimize Zn binding and substrate hydrolysis. The resultant peptides were synthesized and assessed for Zn and substrate binding affinity by fluorescence and ITC followed by evaluation of catalytic efficiency with UV-based enzyme kinetic assays. We were successful in mimicking carbonic anhydrase activity in a peptide of twenty two residues, using p-nitrophenyl acetate as a CO_2_ surrogate. Although our design had modest activity, being a simple structure is an advantage for further improvement in efficiency. Our approach opens a way forward to evolving an efficient biocatalyst for any industrial reaction of interest.

## Introduction

Polypeptides fold and adopt three dimentional structures suitable for specific functions. The physical basis of protein folding as a function of amino acid sequence remains unclear.[1] One approach to understanding the basis for folding is protein design, which tests our knowledge of molecular determinants of structure, stability and function. Designs are approached computationally, by a so called inverse approach,[2], [3], [4], [5], [6], [7] a method that has matured since Mayo's redesign of a Zn-free protein into a Zn-finger fold. Artificial proteins have since been created, and natural proteins have been retrofitted to possess desired functions as sensors and enzymes.[8], [9], [10], [11], [12] These successes probably reflect the success of protein evolution by optimizing sequences to introduce novel activity on pre-existing folds. The independence of evolution of function from evolution of fold-structure suggests a design approach in which first folds-structure and then sequences over the folds may be designed to incorporate specific activity independently.

Recent advances in protein design have motivated researchers to design enzymes capable of catalyzing a wide range of reactions of biotechnological and pharmaceutical interest. Design of an enzyme from scratch provides a way to dissect the elements that contribute to folding and catalytic activity.[13] However, designing an efficient enzyme from first principles remains an unrealized ambition because such a goal requires design of a protein with a catalytic apparatus specific for the desired reaction. Consideration of stabilization of the transition state, release of products, restoration of the active site and associated conformational changes in protein strcture makes the design a formidable challenge.[14], [15], [16], [17] The design of an artificial enzyme can be approached either through de novo design, wherein both protein topology and the active site are built from scratch, or by grafting the active site onto a large number of protein scaffolds of known three-dimensional structure. Although the design of an artificial enzyme is a promising area of research, but still this field is in infancy. Valuable progress have been made in the development of artificial enzymes that catalyzes a retro-aldol reaction and kemp elimination reaction.[18], [19], [20], [21] One recent report provided an elegant de novo design of an enzyme mimic of carbonic anhydrase with full structure details, which is quite encouraging.[22]

In the present study, we used a de novo design approach to evolve an artificial enzyme possessing carbonic anhydrase activity. Because natural carbonic anhydrase is a highly efficient enzyme, it has been identified as a promising candidate for biological sequestration of CO_2_.[23] We set out to design an artificial metallo-hydrolase having an αββ topology based on a Zn-finger fold. We then grafted the catalytic site Zn(His)_3_O onto the base and the substrate binding site onto the rim of the funnel of an αββ protein. We shuffled the residues in substrate binding site and linker loop to dissect different factors responsible for fold stability, substrate binding and catalysis. The designed sequences were then synthesized and characterized for Zn binding, substrate binding, and hydrolase activity using various spectroscopic tools. Our designed Zn finger-based hydrolase is a primordial enzyme candidate with modest activity, and being significantly simpler in structure, has the potential for further improvement in catalytic efficiency for wider application.

## Results

### Design

The design of a 21-residue αββ protein having Zn tri-coordinated in an active site cleft for possible application as a hydrolase enzyme has been reported.[24] We have assessed this protein for the impact of structure modification on catalysis. Design of variants was approached with a two-step algorithm, design of molecular fold structure and sequence optimization to achieve activity. Design of molecular fold was approached with α-helix and β-hairpin folds and involved their organization into tertiary structure over a connector of three to four residues. The connector was examined for effects of modifying its length and structure. Zn was incorporated for ordering of tertiary structure and organization of the catalytic apparatus by coordination to a triad of His residues in a molecular pocket of the αββ fold. The catalytic Zn complex was chosen to be at the base of a funnel-shaped pocket and side chains suitable for substrate binding were placed on the rim of the funnel. The β-hairpin building block of the protein was nucleated over a ^D^Pro_17_–Gly_18_ dipeptide capable of adopting a Type II' β-turn structure. The α-helix building block of the protein was nucleated over an Ac-^D^Pro_1_- ^L^Gln_2_ dipeptide capable of adopting a Type II' β-turn structure and acting as a helix nucleator at its C terminus. The connector joining the α-helix and β-hairpin modules was provided in positions 11–13 in a 21-resdiue variant protein A1–A3 and positions 11–14 in a 22-residue variant protein B1–B5. The connector may be critical to efficient folding and catalysis, thus we explored the effects of length and other structural variations.

**Figure 1:**
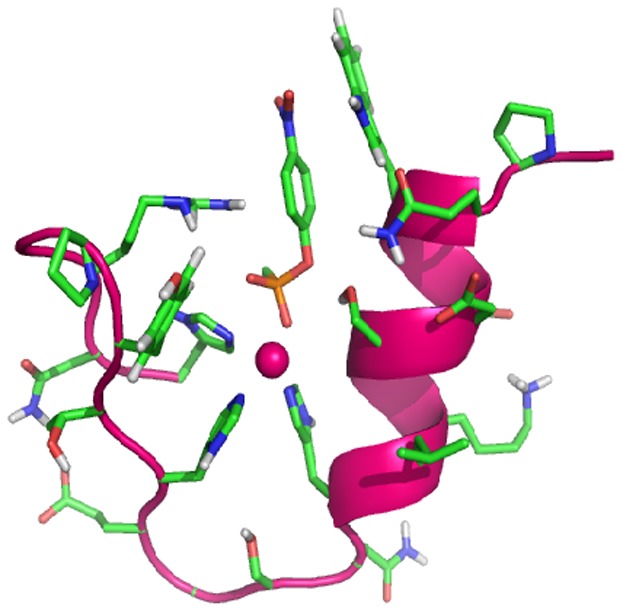
Cartoon representation of computationally designed molecular fold of Zn-finger hydrolase with docked pNPP substrate.

Design of variations in connector structure was implemented computationally. Simulated annealing searches of optimal conformation in connectors of specific length were implemented with NMR-structure determination software CYANA.[25] The broad methodology follows a protocol published from this lab.[26] The connector is initially taken as three and four glycine residues in an extended conformation. Helix and hairpin elements of the targeted protein are constrained in φ,ψ values to the range suitable for modeling the desired secondary structures, helix and hairpin motifs. The constraints are applied with tolerance to provide for sufficient plasticity for packing of α-helix and β-hairpin motifs into globular structures. Three histidines placed appropriately were constrained to the geometry suitable for their coordination of a Zn atom. Polypeptides with Leu residues in every sequence position except those already defined were submitted to repeated cycles of simulated annealing with CYANA. The structures recovered from independent runs were analyzed in glycines of linker structures in their statistics of distribution in Ramachandran φ,ψ space. Based on the preferences noted in specific glycines, the residues were targeted for replacements with suitable side chain-bearing residues of L or D structure. One variant each in the 21-residue protein A and the 22-residue protein B was left with glycine linkers. Other variants were provided with amino acid residues in DLDD and LDLD stereochemical combinations as noted in Table 1. Sequence designs were implemented by the inverse application of protein side chains based on our physico-chemical intuition required for fold stability and function. Amino acids were applied suitably for substrate binding over a combination of neutral-aromatic and cation-aromatic side chains. Position specific mutants were prepared to assess effects on substrate binding or catalytic function. Thus, Tyr_2_ and Gln_2_ were considered as alternative residues for hydrogen bonding with the nitro group of pNPP/pNPA and assistance in substrate recognition. Gln_4_ was mutated to Glu_4_ to create a Glu_4_-Lys_8_ (i-i+4) salt bridge for possible helix stabilization. An Ala_5_ to Asp_5_ mutation was chosen to test its possible involvement in the catalytic apparatus. Results of simulation suggested the possibility of Asp_7_ interfering with Zn coordination, a possibilty that was tested by mutation to Ala_7_. An Arg_9_ to Leu_9_ mutation was selected for assessment of effects on conformation and solubility. Arg_19_ in proteins A1-A3 was moved to Arg_20_ in proteins B1–B5 for possible electrostatic interaction with the transition state analog pNPP to test for a possible effect on catalysis of hydrolysis. Trp_2_ and Tyr_17_ were included for possible π-π interactions with pNPP/pNPA. Gln_16_ and Tyr_2_ were swapped for better steric compatibility with Trp_3_ and Tyr_20_. The sequences of mutants in the two protein families A and B are given in Table 1, and a cartoon representation of one of the designed variants is presented in Figure 1.

**Table 1:** Sequences of designed variants of Zn-Hydrolase.

	Sequences
**A1**	Ac-^D^Pro_1_-Tyr_2_-Trp_3_-Gln_4_-Ala_5_-Ser_6_-Asp_7_-Lys_8_-Arg_9_-His_10_-^D^Glu_11_-Gly_12_-Asp_13_-His_14_-Ser_15_-Gln_16_-^D^Pro_17_-Gly_18_-Tyr_19_-Thr_20_-His_21_-NH_2_
**A2**	Ac-^D^Pro_1_-Tyr_2_-Trp_3_-Gln_4_-Ala_5_-Ser_6_-Asp_7_-Lys_8_-Arg_9_-His_10_-Gly_11_-Gly_12_-Gly_13_-His_14_-Ser_15_-Gln_16_-^D^Pro_17_-Gly_18_-Tyr_19_-Thr_20_-His_21_-NH_2_
**A3**	Ac-^D^Pro_1_-Gln_2_-Trp_3_-Glu_4_-Asp_5_-Ser_6_-Ala_7_-Lys_8_-Leu_9_-His_10_-Gly_11_-Gly_12_-Gly_13_-His_14_-Thr_15_-Tyr_16_-^D^Pro_17_-Gly_18_-Arg_19_-Asn_20_-His_21_-NH_2_
**B1**	Ac-^D^Pro_1_-Tyr_2_-Trp_3_-Gln_4_-Ala_5_-Ser_6_-Asp_7_-Lys_8_-Arg_9_-His_10_-Gly_11_-Gly_12_-Gly_13_-Gly_14_-His_15_-Ser_16_-Gln_17_-^D^Pro_18_-Gly_19_-Tyr_20_-Thr_21_-His_22_-NH_2_
**B2**	Ac-^D^Pro_1_-Gln_2_-Trp_3_-Glu_4_-Asp_5_-Ser_6_-Ala_7_-Lys_8_-Leu_9_-His_10_-Gly_11_-Gly_12_-Gly_13_-Gly_14_-His_15_-Thr_16_-Tyr_17_-^D^Pro_18_-Gly_19_-Arg_20_-Asn_21_-His_22_-NH_2_
**B3**	Ac-^D^Pro_1_-Gln_2_-Trp_3_-Glu_4_-Asp_5_-Ser_6_-Ala_7_-Lys_8_-Leu_9_-His_10_-Gly_11_-Gly_12_-Gly_13_-Gly_14_-Asp_15_-Thr_16_-Tyr_17_-^D^Pro_18_-Gly_19_-Arg2_0_-Asn_21_-His_22_-NH_2_
**B5**	Ac-^D^Pro_1_-Gln_2_-Trp_3_-Glu_4_-Asp_5_-Ser_6_-Ala_7_-Lys_8_-Leu_9_-His_10_-^D^Asn_11_-Ser_12_-^D^Ala_13_-^D^Glu_14_-His_15_-Thr_16_-Tyr_17_-^D^Pro_18_-Gly_19_-Arg_20_-Asn_21_-His_22_-NH_2_
**B4**	Ac-^D^Pro_1_-Gln_2_-Trp_3_-Glu_4_-Asp_5_-Ser_6_-Ala_7_-Lys_8_-Leu_9_-His_10_-Asn_11_-^D^Ser_12_-Ala_13_-^D^Glu_14_-His_15_-Thr_16_-Tyr_17_-^D^Pro1_8_-Gly_19_-Arg_20_-Asn_21_-His_22_-NH_2_

### Synthesis and characterization

The identities of the peptides, synthesized manually with solid-phase chemistry, were confirmed with mass sprctrometry and NMR. Spectra are shown in Figure S1–S2 in File S1. Ion peaks corresponding to expected molecular masses appear in MALDI-MS. ^1^H NMR spectra recorded in 90∶10 H_2_O:D_2_O mixture at pH 7 were sharp and well-dispersed in resonances. No notable effects of dilution were observed in the spectra recorded at 2.5 and 0.25 mM peptide concentrations (data not shown). All peptides were freely soluble and, considering the lack of a dilution effect in NMR, they appeared to be devoid of aggregation. Based on NMR spectra, some of the peptides may have been contaminated with trace impurities.

For peptide B2, we observe anomalous CD patterns. We did not observe well defined –ve band at 222 nm of α-helix which could presumably due to contribution of +ve band of aromatic exiton couplet which masks the polypeptide backbone absorbance. This couplet is normally evidence for interacting aromatic groups.[27], [28] Tyr_16/17_ may have been interacting with Trp_3_, implying that the structure was folded to place the aromatics in close proximity.

### Binding of Zn^2+^ ion

**Figure 2:**
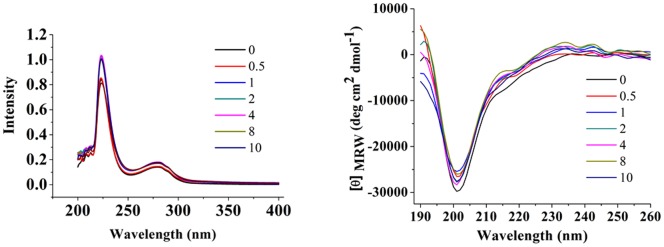
Titration of peptide B2 with Zn^2+^ in Tris-HCl buffer(pH 7.5, 20 mM) at 25°C. UV absorbtion spectral traces (left panel); and CD spectral traces (right panel) of peptides on progressive increase of mole ratio of Zn^2+^ added w.r.t. peptide. The mole ratio of added Zn^2+^ added w.r.t. peptide are indicated in legends and the corresponding spectral traces are color coded accordingly.

**Figure 3:**
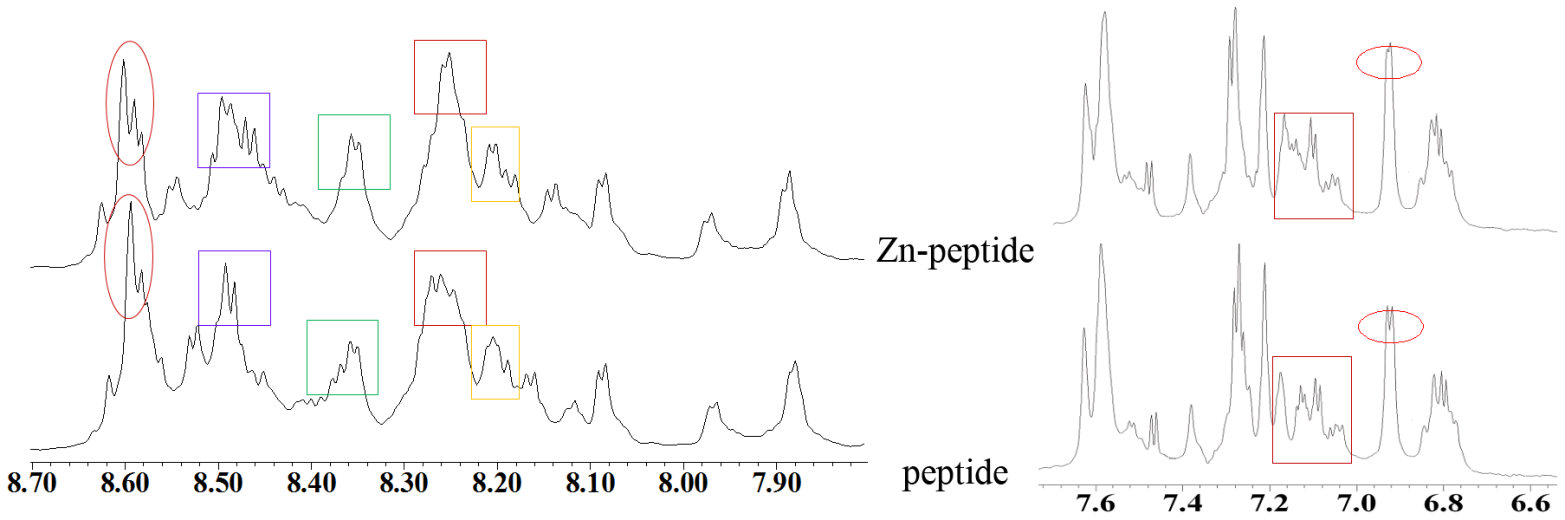
Purturbation of ^1^H-NMR chemical shift of amidic protons (left side) and aromatic protons (right side) of B2 peptide in presence and absence of Zn^2+^ ion in water.

**Figure 4:**
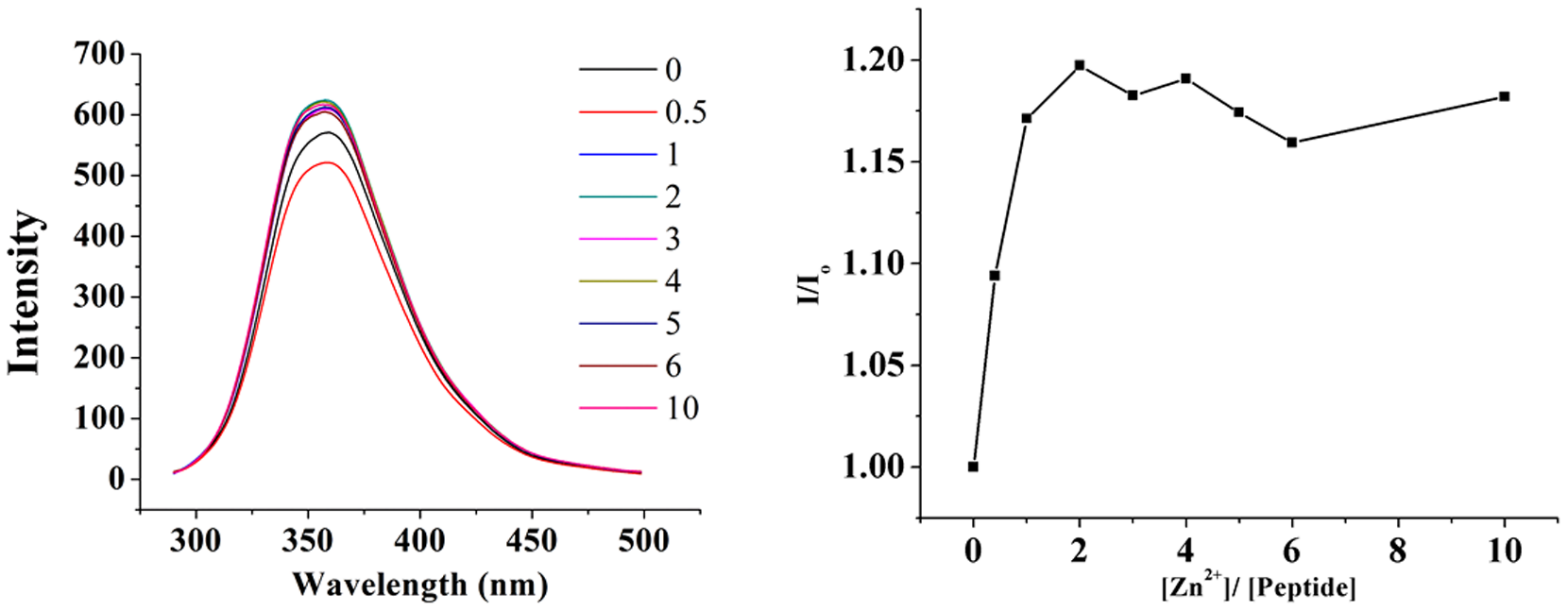
Fluorescence spectra obtained during the titration of peptide B2 (20 µM) with increase concentration of Zn^2+^ in Tris-HCl buffer (pH 7.5, 20 mM) at 25°C (left panel). The concentration of Zn^2+^ in solution are indicated in legends and the corresponding spectral traces are color coded accordingly. The plot of relative fluorescence intensity (I/I_0_) as a function of [Zn^2+^]/[peptide] molar ratio displaying saturation at one equivalent of Zn^2+^ ion (right panel).

**Figure 5:**
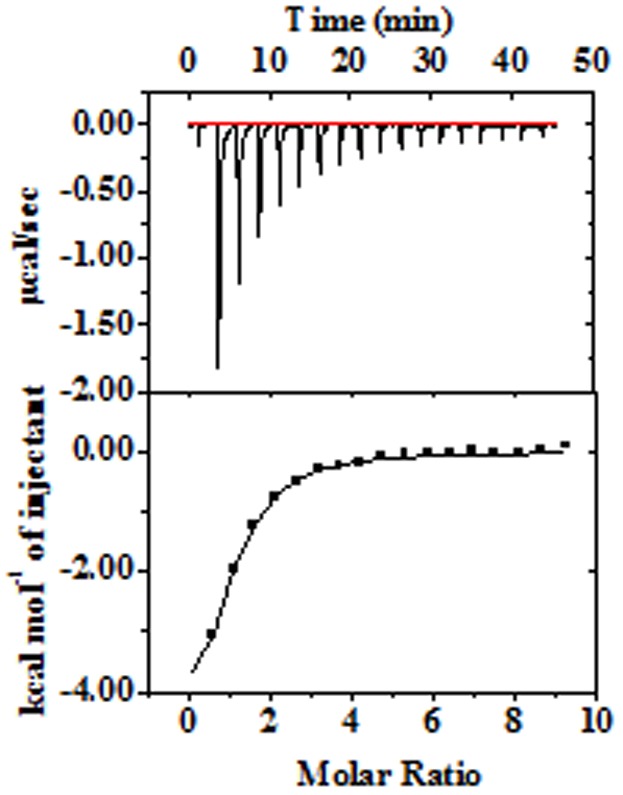
Isothermal calorimetric evaluation of Peptide-Zn^2+^ interactions of B2 variant (100 µM) with Zn(ClO_4_)_2_ (5 mM) in Tris-HCl buffer(pH 7.5, 20 mM) at 25°C. Measured heat change on adding aliquotes of Zn(ClO_4_)_2_ into peptide solution (Upper panel). Integrated heat change in each step with solid line in correspondence of single-site binding model (Lower panel).

The interaction of the Zn^2+^ ion with peptide **B2** was monitored with couple of biophysical techniques, and the results are reported in Figures 2, 3, 4. As per NMR results (Figure 3), perturbation of several NH chemical shifts was observed upon addition of a 5-fold molar excess of Zn(ClO_4_)_2_. The interaction was also monitored with CD, UV, and fluorescence. The results in Figures 2 and 4 indicated that the peptide bound the Zn with a defined stoichiometry; presumably Zn formed a 1∶1 complex with the peptide. Absence of any noteworthy change in spectral properties of the protein suggest two following possibility. One, apo-peptide could be in molten globule state having well defined secondary structure and Zn binding initiate further packing of side chains to adopt native globular structure of Zn-peptide complex. Second, apo-peptide folds in pre-ordered native conformation and Zn binding may not have a role in folding of peptide. Interaction of peptide **B2** with Zn was monitored with ITC. The interaction was saturable and exothermic (Figure 5). Fitting the data to a single site-binding model gave N of 0.94±0.05, K_d_ of 42.9±5.2 µM, ΔH^0^ as −22.9±2.3 kJ/mol, ΔS^0^ as 6.6±0.8 J/mol and ΔG^0^ as −24.9±2.7 kJ/mol. The tight binding of Zn implies that the interaction involved coordination with the histidines.

### Binding with p-nitrophenyl phosphate

**Figure 6:**
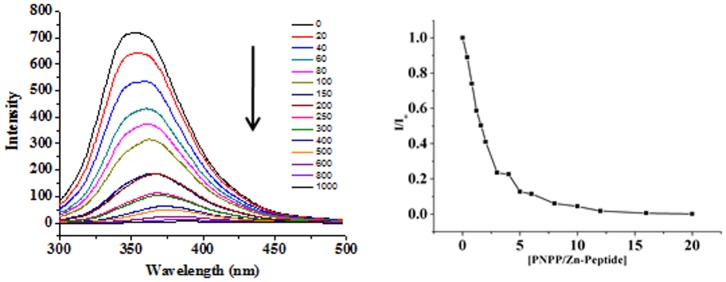
Quenching of tryptophan fluorescence of in situ assembled Zn^2+^-Peptide complex of peptide B2 (20 µM) in Tris-HCl buffer(pH 7.5, 20 mM) at 25°C on progressive titration with increasing pNPP concentration (0–400 µM) (left panel), and plot of relative fluorescence intensity as a function of [pNPP]/[Zn-peptide] molar ratio (right panel).

The peptides were evaluated for binding with *p*-nitrophenyl phosphate (*p*NPP). The interaction was tested against proteins ligated and unligated with Zn. On titration with pNPP, all sequences manifested quenching of Trp fluorescence (Figure 6 and Figure S3 of File S1), with an ∼30 nm red shift of emission on interaction with pNPP. The binding energies of pNPP with apo-peptides and their Zn-peptide complexes were determined from fluorescence quenching data using the Stern-Volmer equation (see Table 2). The binding energies for apo-peptides and their Zn-peptide complexes were comparable and varied within a narrow range. The modeled proteins were tested for ligand binding with Autodock (Table 2). The computationally determined energies were similar to each other and somewhat smaller than those observed experimentally.

**Table 2:** Binding energy of pNPP with variants of free peptide and Zn-peptide complex determined with fluorescence and AutoDock.

Variants	Binding Energy (Kcal/mol)		
	Fluorescence		AutoDock
	Peptide	Zn-Peptide	Zn-Peptide
**A1**	−6.73±0.48	−7.03±0.32	−3.83
**A2**	−6.85±0.25	−7.06±0.16	−4.11
**A3**	−5.84±0.67	−6.27±0.42	−4.67
**B1**	−5.75±0.86	−6.26±0.52	−3.65
**B2**	−6.05±0.19	−6.85±0.25	−4.42
**B3**	−6.50±0.36	−6.65±0.17	−3.51
**B4**	−6.36±0.45	−6.86±0.14	−3.12
**B5**	−6.25±0.78	−7.89±0.28	−4.41

### Evaluation of Catalysis

**Figure 7:**
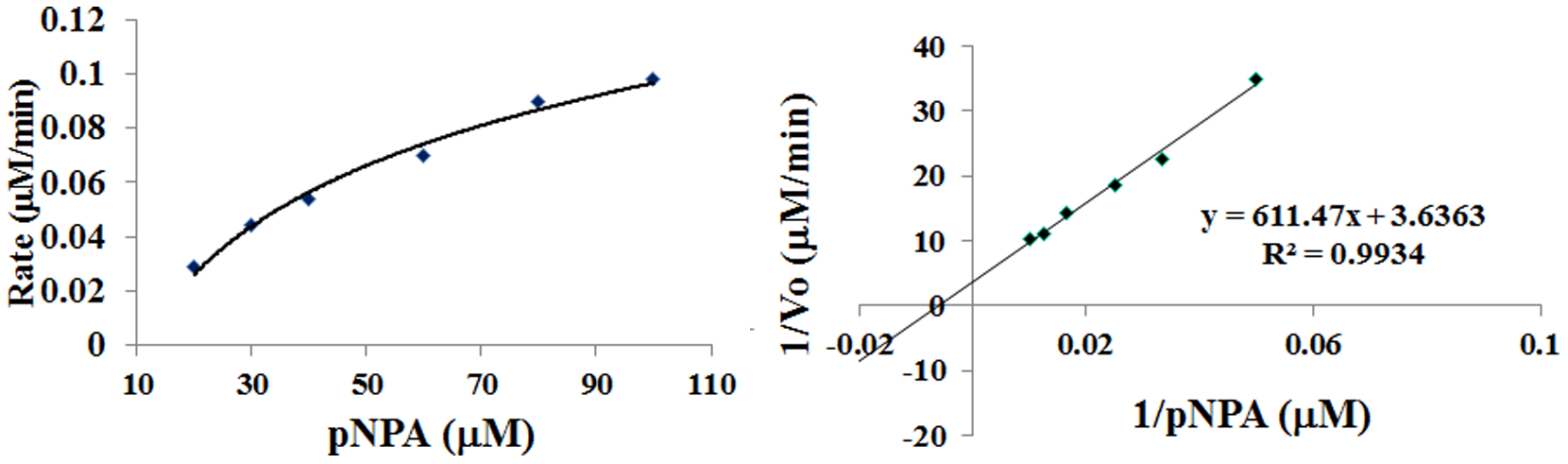
UV monitored rate of hydrolysis of pNPA with in situ assembled Zn-peptide complex in Tris-HCl buffer(pH 7.5, 20 mM) at 25°C on progressive titration with increasing pNPA concentration (10–100 µM) in the form of Michalies-Menten plot (left panel) and Lineweaver-Burk plots (right panel).

Zn-peptide complexes were evaluated for hydrolase function against p-nitrophenyl acetate (pNPA). All in situ assembled metal-peptide complexes hydrolyzed pNPA. Representative results in Figure 7 indicate a tendency towards saturation kinetics with increasing substrate concentration. The data for all proteins gave linear Michaelis-Menten plots (Figure S4 in File S1). The derived kinetic constants are summarized in Table 3. We have also performed enzymatic assay of apo-peptide for some of varients and results are summarized in Figure S5 and Table S1 of File S1. We observed that apo-peptides display very low catalytic efficiency compared to their in-situ assembled Zn-peptide complexes.

**Table 3:** Michaelis-Menten kinetic parameters of Zn-peptide complexes of variants derived for the hydrolysis of pNPA.

Variants	Kinetics Rate		
	*10^−5^* K_M_ (M)	*10^−5^* K_Cat_ (s^−1^)	K_Cat_/K_M_ (M^−1^ s^−1^)
**A1**	12.30±2.68	21.40±4.44	1.74±0.07
**A2**	9.52±7.02	9.58±5.11	1.12±0.23
**A3**	8.14±5.62	12.00±8.23	1.52±0.21
**B1**	12.02±6.23	17.40±6.55	1.58±0.39
**B2**	3.82±2.04	8.80±2.79	2.50±0.67
**B3**	33.82±21.07	86.37±40.73	2.85±0.40
**B4**	19.83±9.56	28.70±15.20	1.43±0.06
**B5**	52.02±29.54	68.50±35.01	1.60±0.32

All proteins were comparable in catalytic attributes. There were marginal effects of the diverse structure variations on kinetic properties of the enzymes. **A3** and **B2** manifested slightly better turnover compared to **A2** and **B1**. **B2** and **B3**, having four glycine linkers, gave better turnover than **B4** and **B5**, which had more rigid linkers. Furthermore, a four-glycine linker in **B2** and **B1** gave a better turnover number than the three-glycine linker in **A3** and **A2**. All peptides had comparable catalytic proficiency.

## Discussion

Our objective in this study was to design a minimal carbonic anhydrase mimic using a de novo protein design approach. We followed a step-by step design of first folding the main chain followed by sequence variation for substrate binding and catalysis. For optimizing the structure of molecular fold, we designed an αββ protein based on Zn-finger folds. CYANA turned out to be a rapid, high throughput tool for fold optimization. We then inverse-designed the sequences to provide stability to the folds along with providing flexibility of linker regions to optimize Zn binding and substrate hydrolysis. We incorporated Zn(His)_3_O as a catalytic apparatus to mimic carbonic anhydrase, wherein a Zn-bound hydroxyl ion serves as a nucleophile.[29], [30], [31], [32], [33] The substrate-binding pocket was designed using pNPA as a surrogate ester substrate for CO_2_. Because carbonic anhydrase hydrolyzes pNPA and CO_2_ by a similar mechanisms,[34], [35] and pNPA is a chromogenic reporter of hydrolysis, pNPA is a practical substrate to use in a high throughput evolutionary search for variants with hydrolase activity. Also, pNPP is an interesting aid for this approach; as a transition state analog for hydrolysis of pNPA, pNPP provides a selection method involving direct binding with the intended enzymes and basis for testing binding affinity.

Designed structures varied in diverse elements of sequence and stereochemistry were tested for effects on folding and catalytic function by analyzing binding with pNPP and hydrolysis of pNPA. While binding of pNPP and hydrolysis of pNPA were observed, the properties were surprisingly unaffected by the structure variations tested. The results of titration experiments with Zn(ClO_4_)_2_ using different techniques proved that Zn bound to the peptides in a 1∶1 stoichiometry with good affinity. The results of fluorescence quenching experiments demonstrated the substrate binding. The similar substrate affinities of apo-peptides and Zn-peptide complex advocate for the possibility of pre-ordered state of apo-peptide. Although this result support pre-ordered state of apo-peptide, but we can't rule out the possibility of Zn binding assisted transition of molten globule to native conformation. Further experimental evidence needed to understand this phenomenon that can further help in evolving better catalyst. The catalytic efficiencies of all the variants were roughly similar, suggesting that the shuffling of aromatic residues in the active site pockets has no consequence on activity. The observed rates were similar in magnitude to those reported for a previously designed carbonic anhydrase mimic. However, k_cat_/K_M_ varied narrowly, but the magnitudes observed are as per our design consideration. We reshuffled aromatic residues in substrate binding pocket for better substrate binding and hence catalysis. The observed rates of A3 and B2 are better that of A2 and B1 respectively. This suggests that reshuffling of aromatic residues resulted in improved catatalytic efficiency. Catalytic efficiency of peptides (B1, B2, B3) having four residue glycine linker were observed to be higher than the peptides (A2, A3) having three residue glycine linker. Better efficiency of B2 and B3 compared to B4 and B5 suggests that mutants with glycines linker have better efficiency than the rigid stereochemical linkers. Hence, four residue glycine linker are optimal in providing better conformational plasticity desirable for folding and ligand binding followed by its catalysis.

The observed efficiency of our designed Zn-peptide complex is just over 1000-fold less then that of carbonic anhydrase (CA). However, efficiency is low, it is roughly similar in magnitude to the best known CA mimics, i.e., designed metallo-protein complex at pH 7.5.[22] Furthermore, the efficiency of our Zn-peptide complex is many fold superior than other reported small molecule mimic of CA like macrocyclic amine-Zn complexes.[36], [37], [38], [39], [40], [41] Although our designed Zn-finger hydrolase mimics carbonic anhydrase activity, it is many orders of magnitude less efficient than the natural enzyme. Although our designed hydrolase has activity similar to that reported for an artificial metalloenzyme, it is nonetheless novel in fold and smaller in structure, being built from scratch following a step-by-step evolutionary approach. Being simpler, our design offers a significant advantage in terms of exhaustive exploration of fold space for better shape, and of sequence space for improved CO_2_ hydrolysis. This condition further broadens the scope of the designed enzyme for introduction of a prosthetic group to increase thermostabity, immobilization to solid surface and resistance to other chemical species for industrial implementation.

## Conclusion

We present here the design of an artificial Zn-based hydrolase enzyme as a carbonic anhydrase mimic. We targeted a Zn-finger protein for re-engineering as a carbonic anhydrase mimic. Following step-by-step approach, we first optimizes the structure of molecular fold, then we optimize the sequences to introduce catalytic activity in the designed fold. The experimental results indicate that the peptides strongly bind to Zn and in-situ assembled Zn-peptide complex catalyse the hydrolysis of *p*-nitrophenyl acetate. Although we achieved enzyme-like substrate binding and subsequent hydrolysis, catalytic efficiency was poor compared with the natural carbonic anhydrase. Nevertheless, being simple in structure offers an advantage for further improvement in efficiency. The success of this effort is not in terms of the end product, but as a way forward to evolve efficient biocatalysts for diverse industrial applications.

## Experimental Section

### Materials

p-Nitrophenylacetate and Zn(ClO_4_)_2_.6H_2_O were from Sigma Aldrich. Fmoc-protected amino acids, reagents for solid-phase peptide synthesis, Rink-Amide AM resin, DMF, methanol, diethylether, dichloromethane, were from Sigma–Aldrich or Novabiochem- Merck.

### Peptide Design

The protein fold was designed using CYANA.[25] We implemented fold design by constraining secondary-structure elements locally and desired tertiary-structure fold globally. While implementing the simulated annealing with CYANA, we imposed distance and torsional angle constraints with sufficient tolerance for plasticity of conformation and flexibility of atomic packing.

### Molecular Docking

A flexible docking algorithm was implemented with AutoDock 4.0.[42] Central members of first microstate obtained by clustering the three aromatic residues over the molecular dynamics trajectory were chosen for modeling the receptor structure in ligand-peptide complex. A genetic algorithm was used for docking. Using an RMSD tolerance of 2 Å, structurally distinct conformational clusters of the ligand were ranked by increasing energy, and the lowest energy is reported as the binding energy.

### Peptide Synthesis

Synthesis was performed on Rink Amide AM resin using standard Fmoc chemistry and HOBt/DIC as coupling reagents.[43] Each coupling, monitored with Kaiser and chloranil tests, typically required about 6 hrs. Deprotections were carried out with 30% (v/v) piperidine-DMF. N-termini were acetylated (-NHCOCH_3_) with Ac_2_O: DIPEA:DMF in 1∶2∶20 ratio. Cleavage of the final polypeptide and deprotection of side chains were achieved together with reagent K (82.5% TFA/5% dry-phenol/5% thioanisole/2.5% ethandithiol/5% water). The product precipitated with anhydrous diethyl ether was lyophilized from 1∶4 H_2_O:^t^BuOH solution as a white powder. Peptide purity was assessed with HPLC over RP-C18 (10 µM, 10 mm×250 mm; Merck) eluting with CH_3_CN\H_2_O (0.1%TFA) 0–100% gradients.

### Mass Spectrometry

Mass spectra were recorded by MALDI-TOF (Matrix Assisted Laser Desorption Ionization-Time of Flight) mode on an AXIMA-CFR Kratos instrument.

### Nuclear Magenetic Resonance Spectroscopy

^1^H NMR spectra of 2.5 mM concentration were recorded on 700 MHz Bruker instrument at 298 K in 90% H_2_O/10% D_2_O in water with DSS as internal standard at pH 6.7. Solvent was suppressed with pre-saturation or WATERGATE sequence, as provided in the Bruker software.

### Circular Dichroism

Circular Dichroism (CD) was recorded on a JASCO J-810 CD spectropolarimeter at 298 K in a 0.2 cm path length quartz cell with a 2 nm bandwidth in the far-UV (190–250 nm) range. Scanning at 100 nm/min with a 1.0 s time constant in 1 nm steps, five scans were averaged after baseline correction for water. Working solutions of 40 µM concentration of peptides were prepared by optical measurements. The observations in millidegrees were converted to molar residue ellipticity [θ_MRW_].

### Spectrofluorometry

Fluorescence was measured on a Perkin Elmer LS-55 spectrofluorimeter equipped with a standard PMT. Data were collected at 298 K in a 1 ml cell, with λ_excitation_ as 295 nm, λ_emission_ in 300–500 range, with 5 nm excitation and emission slits. A scan rate of 100 nm/min in 1 nm steps was used. The working concentrations were 20 µM peptide and 0–400 µM of pNPP, pNPA in Tris-HCl buffer (20 mM, pH ∼7.5). Stern-Volmer constants (K_SV_) for the external quencher i.e. pNPP, were obtained using the following biomolecular quenching equation. 
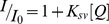
Where I_0_ =  Fluorescence intensity in the absence of external quencher, I =  Fluorescence intensity in the presence of quencher, Q =  Concentration of the quencher, and K_SV_  =  Stern-Volmer constant calculated from the slope of line. The emission maximum intensities of tryptophan were fit as a function of pNPP concentration to the described 1∶1 binding isotherm, and K_d_, hence binding energy, was estimated.

### Isothermal Titration Calorimetry

The calorimetric titrations were performed at 25°C with a microcal isothermal titration calorimeter from MicroCal (Northampton,MA, USA). The sample cell contained peptide at 100 µM, as determined by OD measurements. The reference cell contained Tris-HCl buffer (pH ∼7.5, 20 mM). The 5 mM Zn(ClO_4_)_2_.6H_2_O solution loaded in 250 µL syringe was titrated to the peptide solution in successive additions of 1 µl of Zn(ClO_4_)_2_.6H_2_O with an interval of 150 s. The change in enthalpy (ΔH) due to dilution was determined by titrating Zn(ClO_4_)_2_.6H_2_O into buffer. These backgrounds were subtracted from the ΔH obtained for the corresponding Zn(ClO_4_)_2_.6H_2_O binding to peptide prior to curve fitting. The background-subtracted data were fitted to a model describing a single binding site using the MicroCal software available with the ITC instrument and the binding enthalpy (ΔH°), entropy (ΔS°) and dissociation constant (K_d_) were calculated respectively.

### Enzyme Activity

The kinetics of hydrolysis was monitored spectrophotometrically on a Perkin Elmer spectrophotometer, fitted with peltier, using p-nitrophenylacetate (pNPA) as substrate, by observing the production of the p-nitrophenolate anion at 410 nm. A stock solution of pNPA was prepared in water with a few drops of acetonitrile added to solubilize pNPA. Peptide concentration in the assays was in the range of 20 µM. Hydrolase activity was evaluated in 20 mM Tris-HCl buffer of pH 7.5 at 25°C, by varying substrate pNPA concentration (10–100 µM). The catalyzed rate of pNPA hydrolysis was measured by an initial slope method, following the increase in 410 nm absorption by the *p*-nitrophenolate ion.

## Supporting Information

File S1 Includes Figure S1–S5 and Table S1.
(DOCX)
