# Supplementary material for: Design of a Zinc-Finger Hydrolase with a Synthetic αββ Protein
Source: PLoS One. 2014 May 9;9(5):e96234. doi: 10.1371/journal.pone.0096234 (PMC4015931; doi:10.1371/journal.pone.0096234)
Supplement: File S1 — Includes Figure S1–S5 and Table S1. (DOCX) [file pone.0096234.s001.docx]

**Supporting Information**

**Design of a Zinc-Finger Hydrolase with Synthetic** αββ **Protein**

Kinshuk Raj Srivastava*, Susheel Durani

Department of Chemistry, Indian Institute of Technology Bombay, Mumbai-400076, India

AUTHOR EMAIL ADDRESS: kinshukraj2@gmail.com

|  |  |
| --- | --- |
| **A1** | **A2** |
|  |  |
| **A3** | **B1** |
|  |  |
| **B2** | **B3** |
|  |  |
| **B4** | **B5** |

**Figure S1:** ^1^H-NMR spectra of Zn-Hydrolase peptide variants recorded in H_2_O:D_2_O (90:10%) at 700 MHz.

**A1**


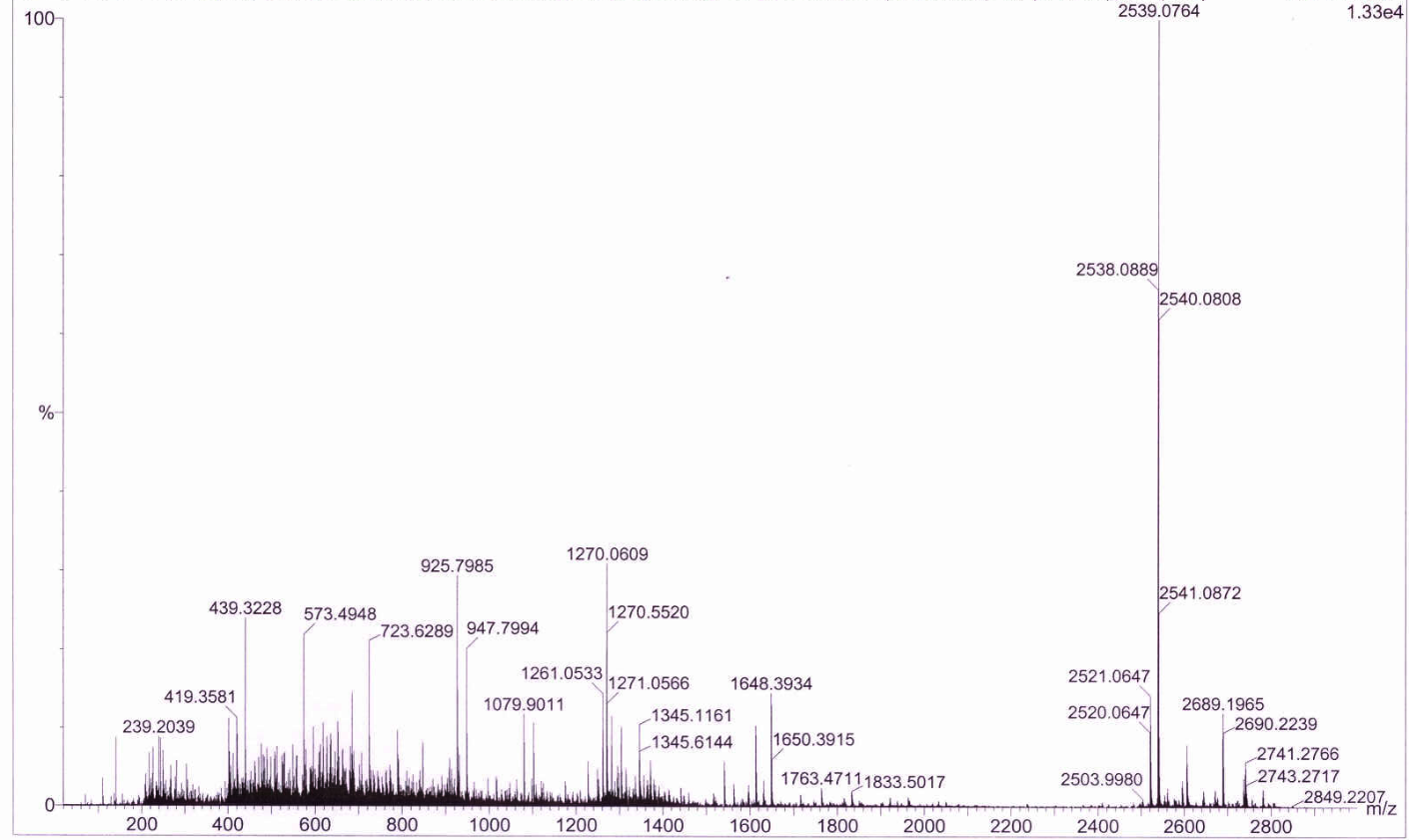


**A2**


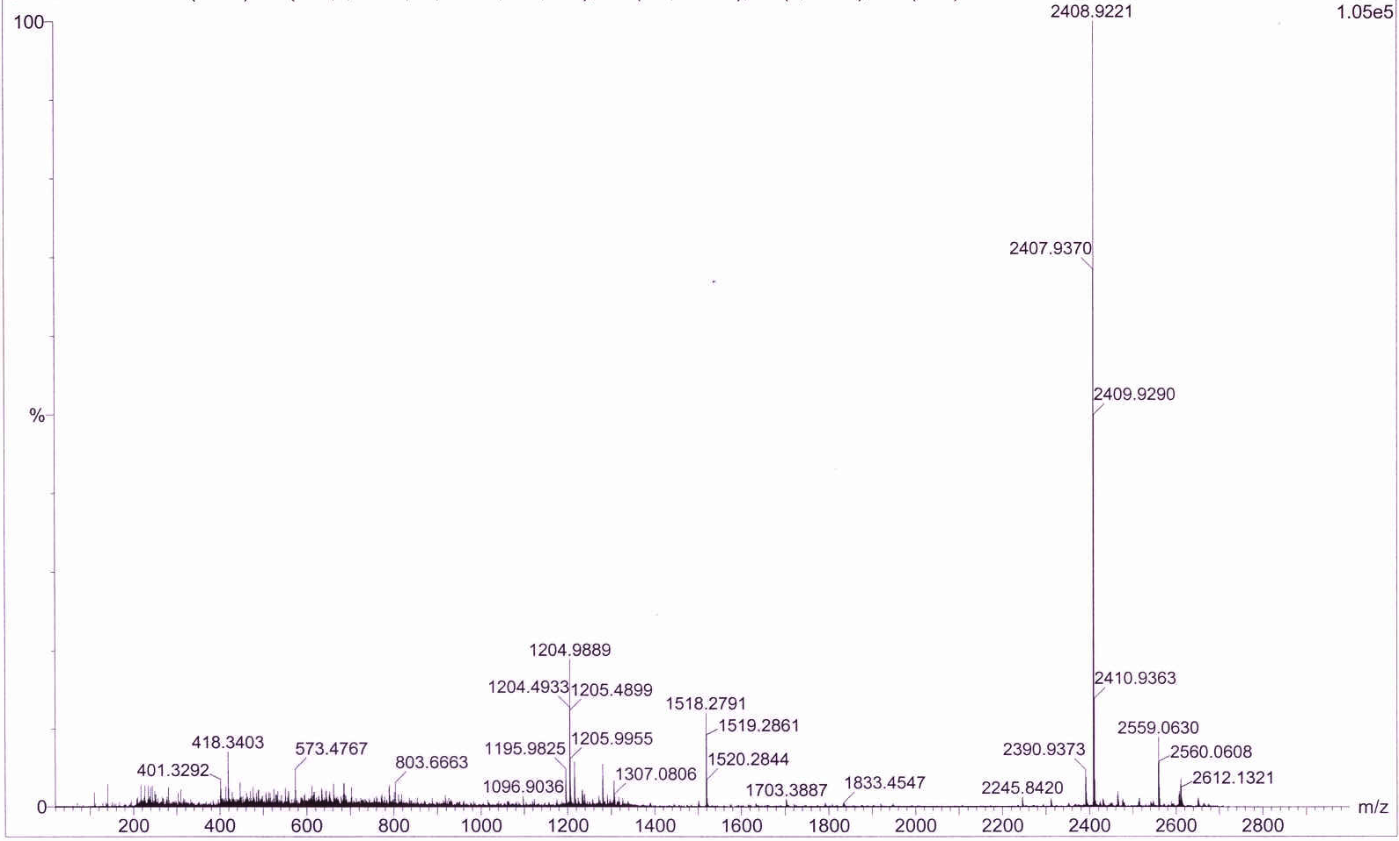


**A3**


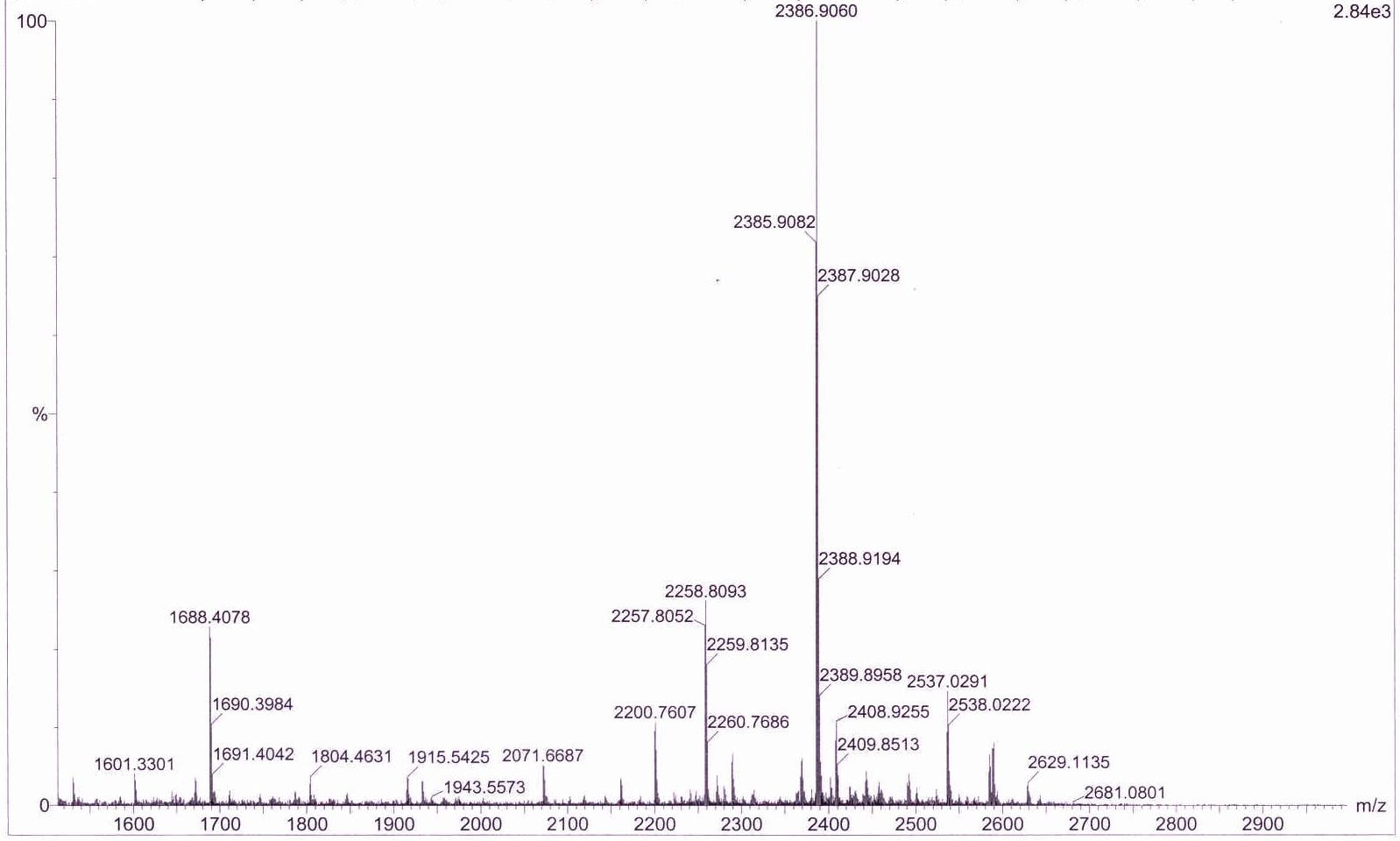


**B1**


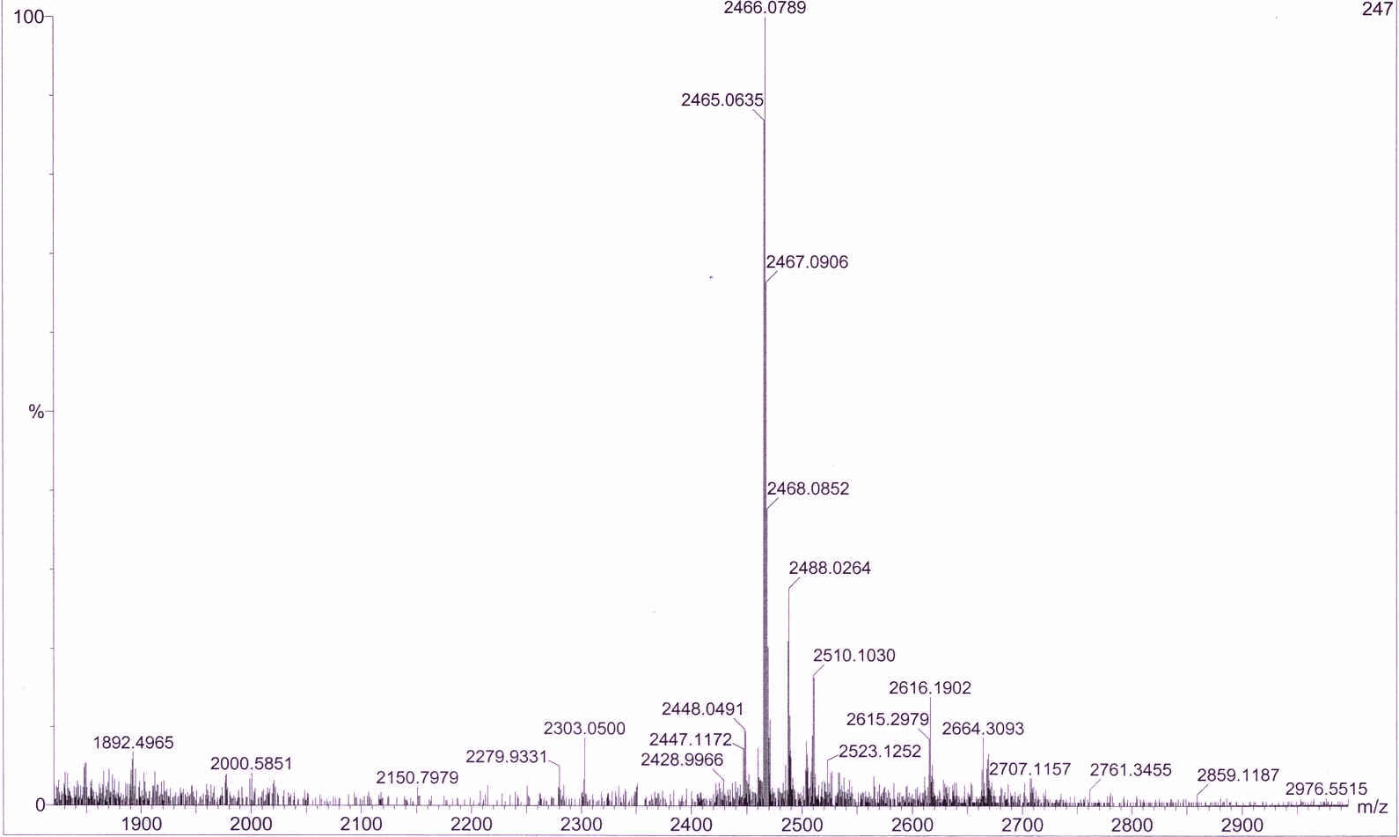


**B2**


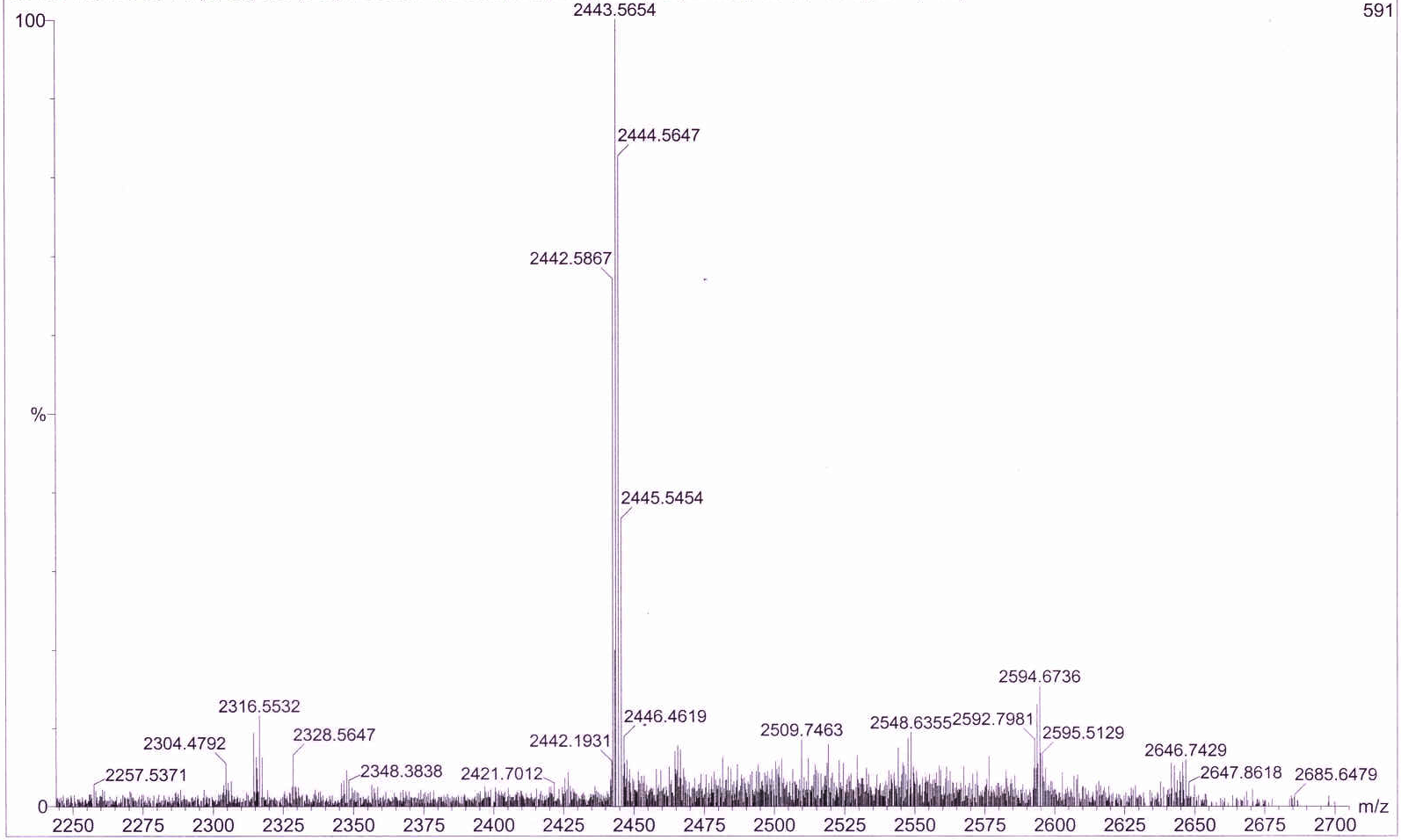


**B3**


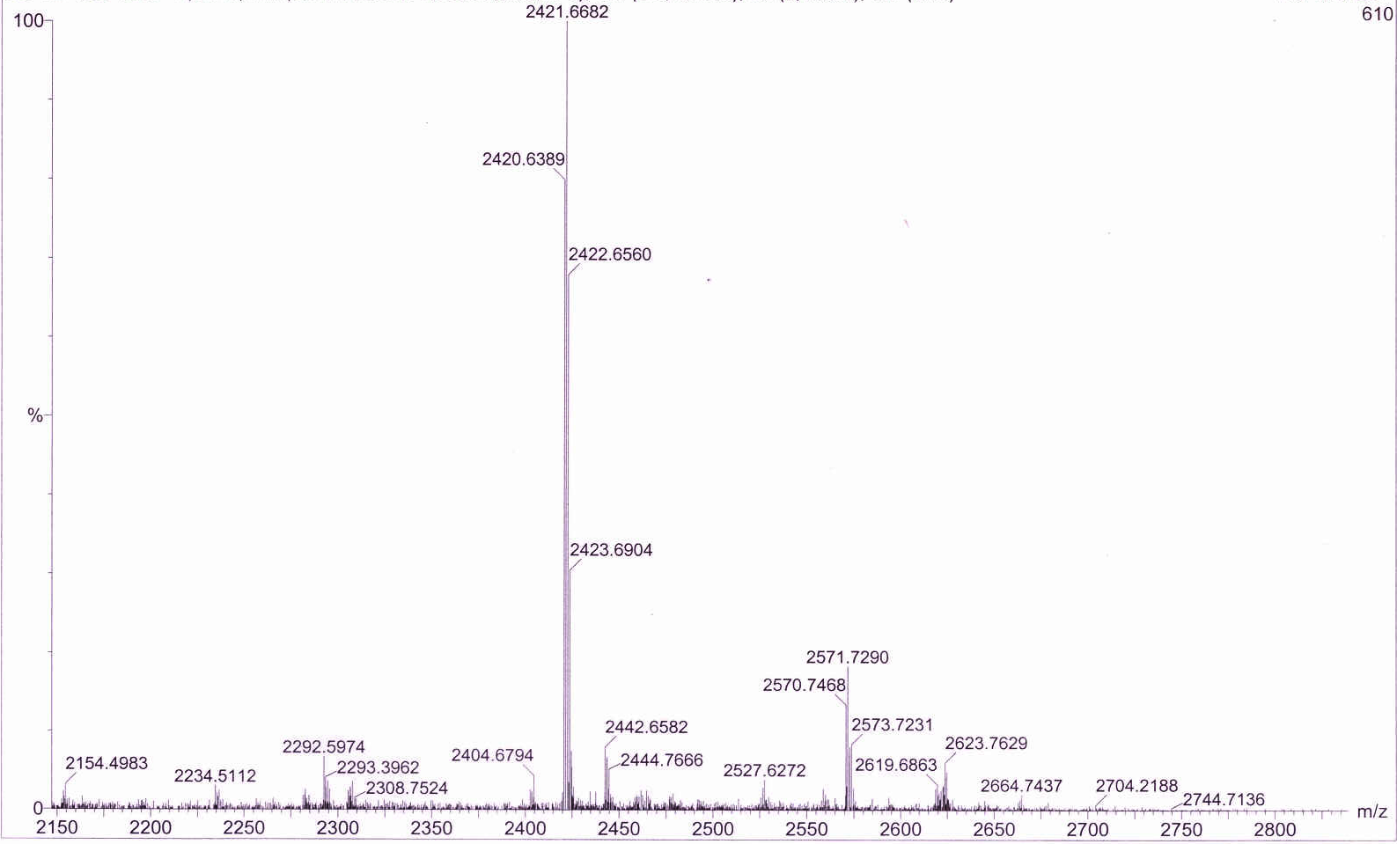


**B4**


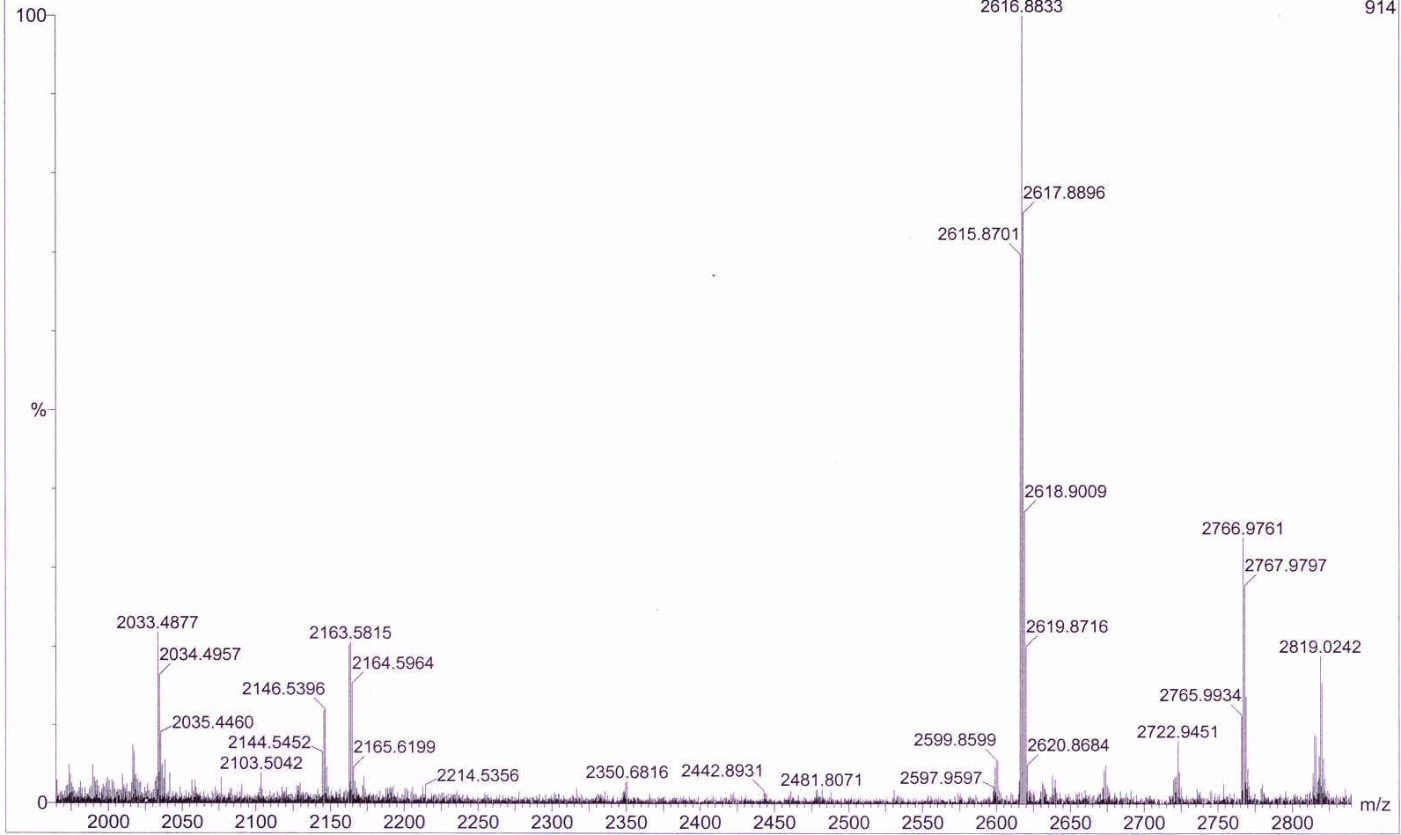


**B5**


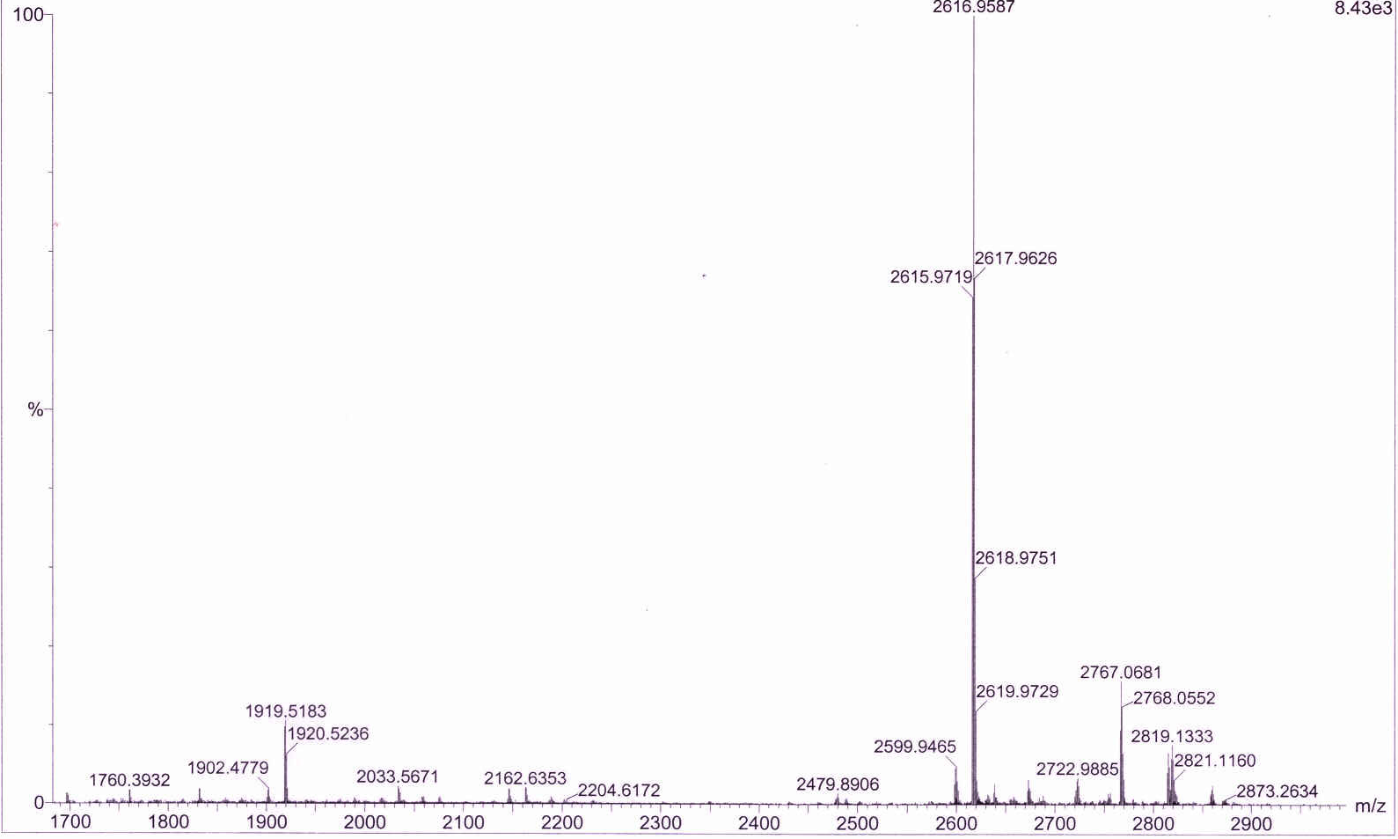
**Figure S2**: ESI-MS mass spectra of Zn-Hydrolase peptides variants.

| **** | **** | **** | **** |
| --- | --- | --- | --- |
| **A1** | **A2** | **A3** | **B1** |
| **** | **** | **** | **** |
| **B2** | **B3** | **B4** | **B5** |

**Figure S3:** Fluorescence spectra during the titration of Zn-Peptide complex of variants (20μM) with increasing concentration of pNPP (μM) displaying the quenching showing the quenching of tryptophan fluorescence on binding to pNPP.

| **Variants** | **Michaelis-Menten Saturation Curve** | **Lineweaver–Burk Plot** |
| --- | --- | --- |
| **A1** | 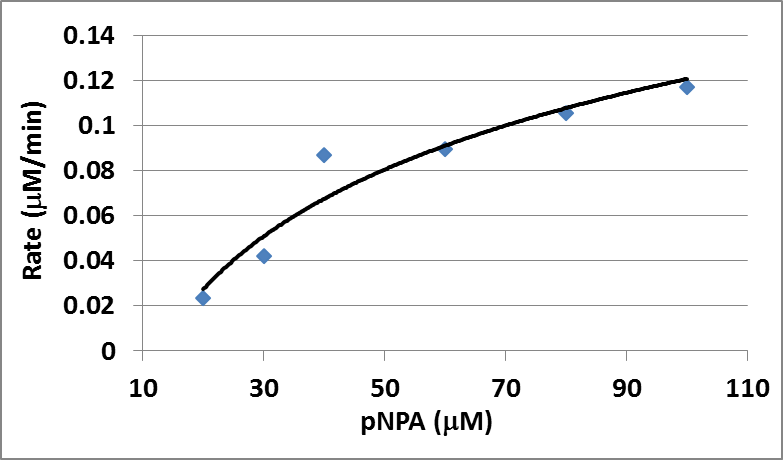 | 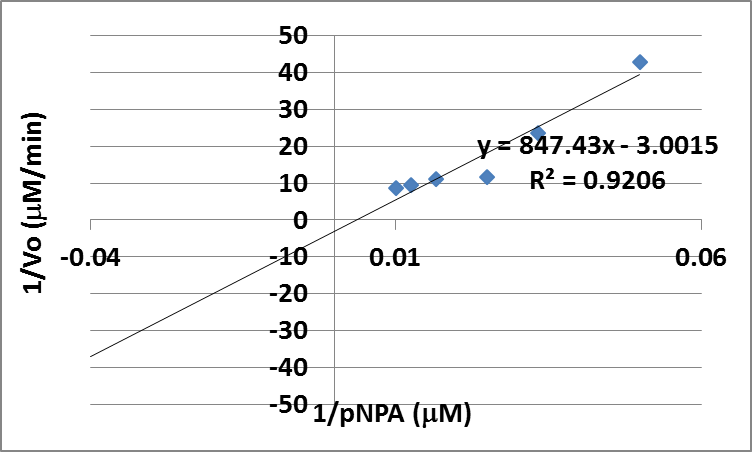 |
| **A2** | 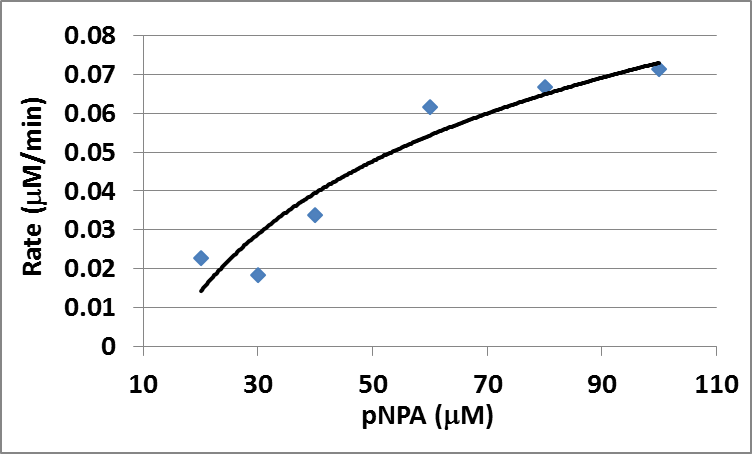 |  |
| **A3** | 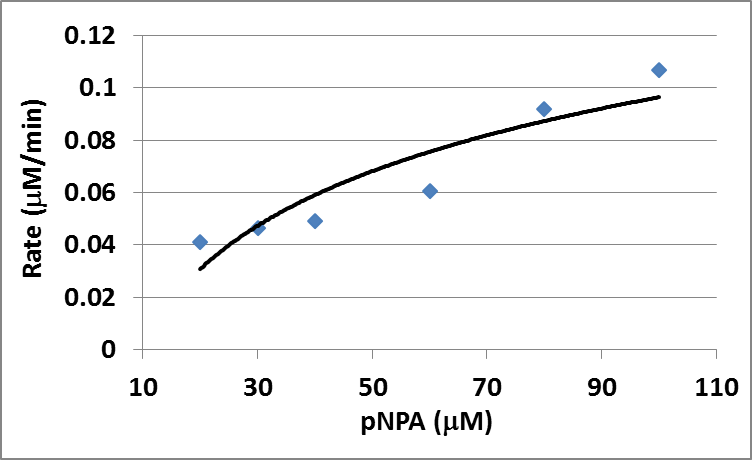 | 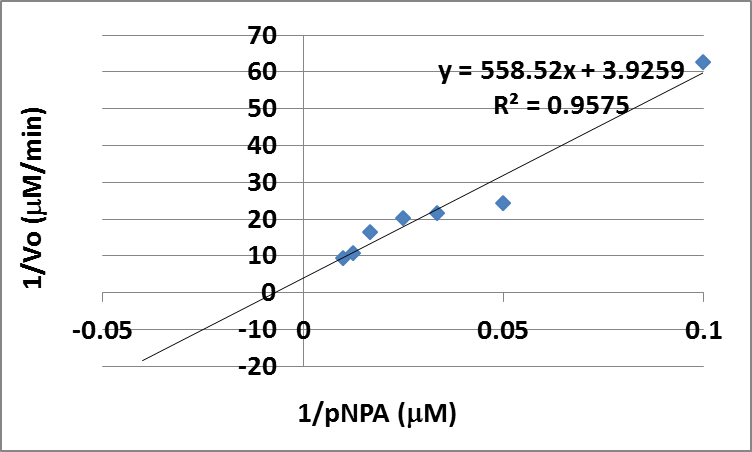 |
| **B1** | 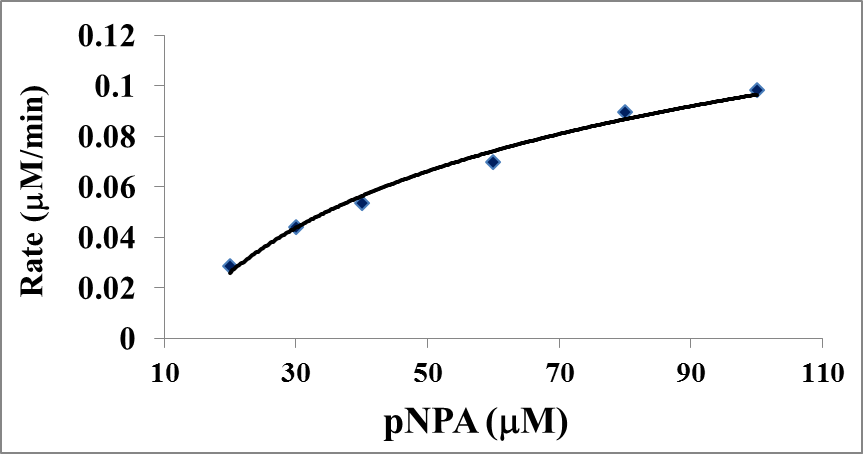 |  |
| **B2** | 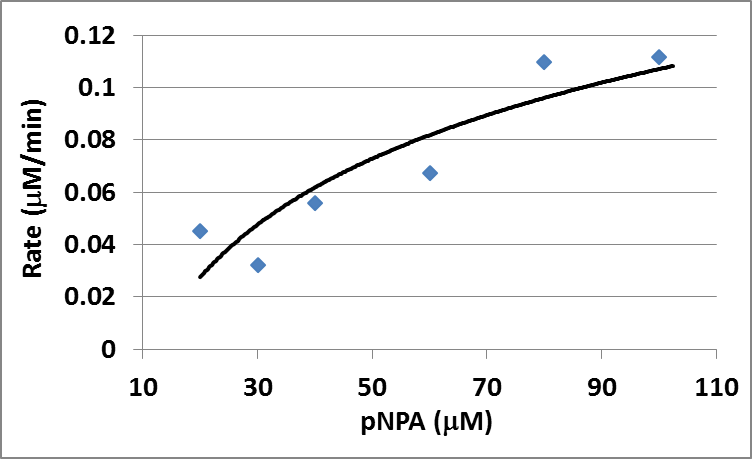 |  |
| **B3** | 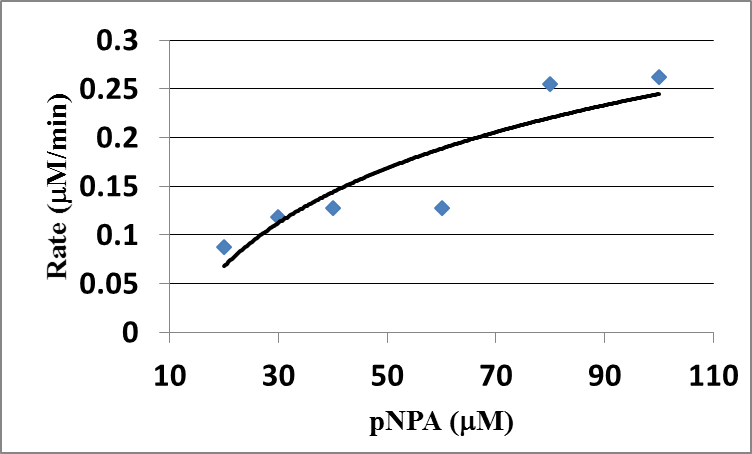 | 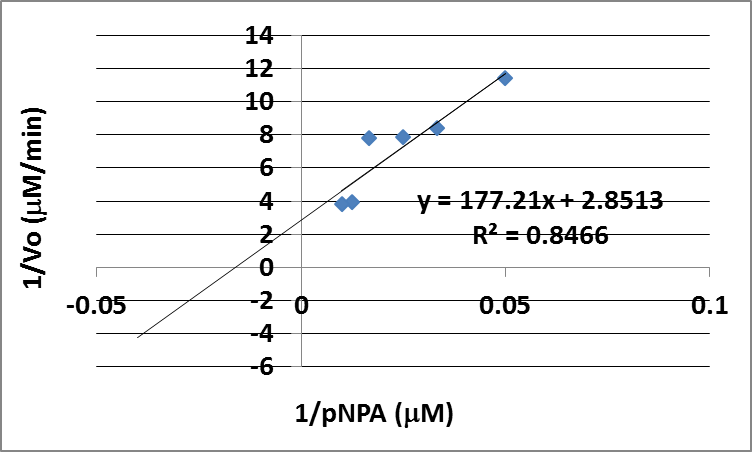 |
| **B4** | 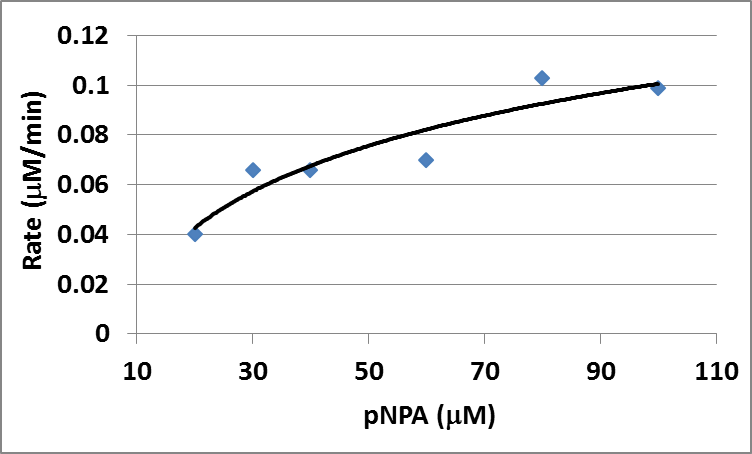 | 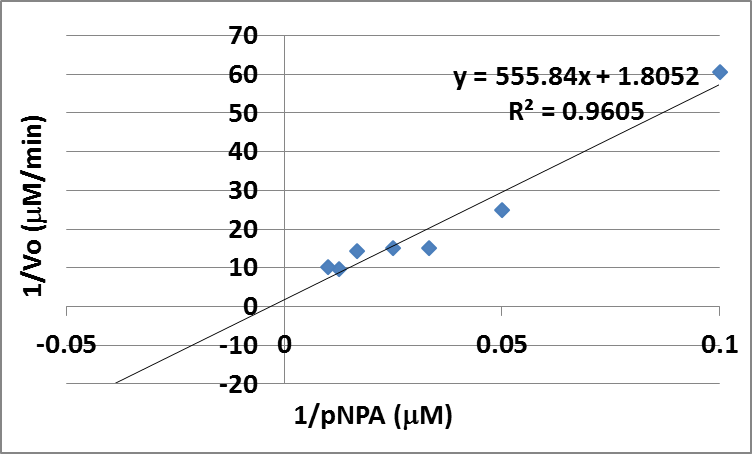 |
| **B5** | 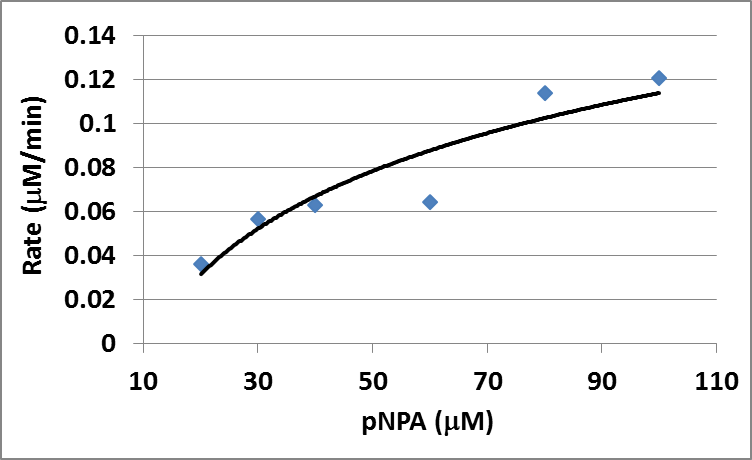 |  |

**Figure S4:** Enzyme Kinetics profiles of the in situ Zn-peptide complex of mutants.

| **A3** | **B1** | **B5** |
| --- | --- | --- |
| 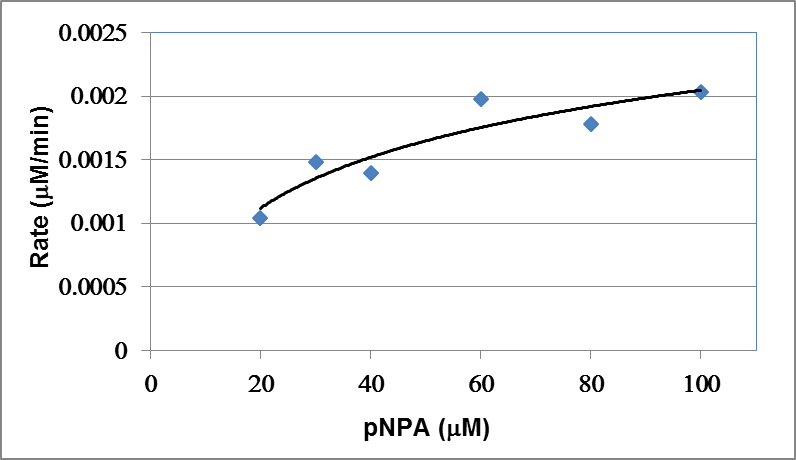 | 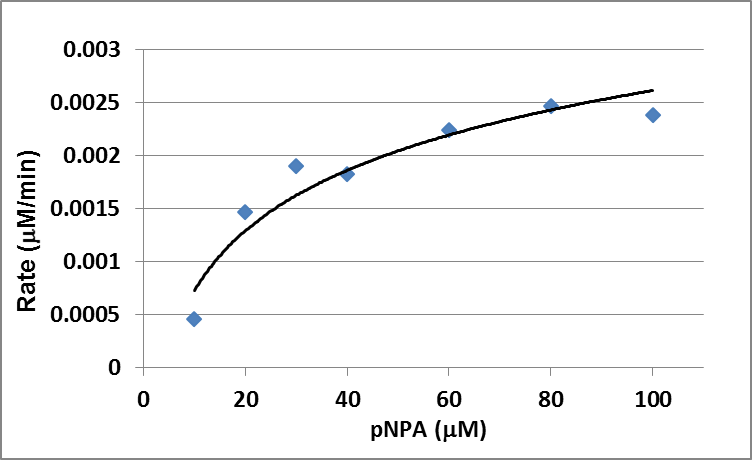 | 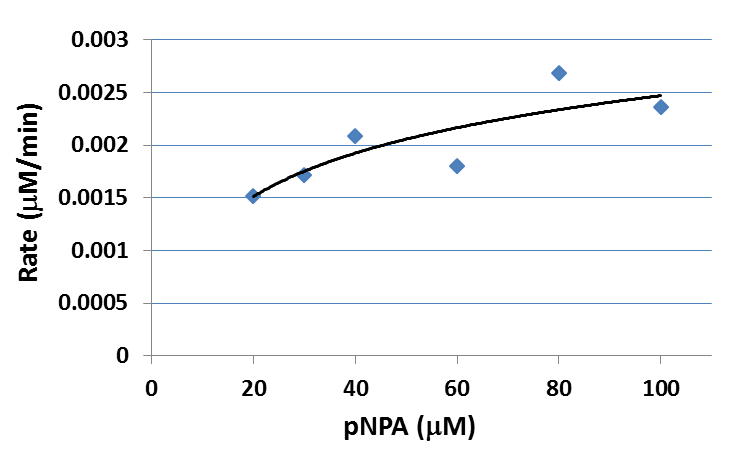 |

**Figure S5:** Michaelis–Menten enzyme kinetics profiles pNPA hydrolysis by peptide mutants only.

**Table S1:** Average catalytic efficiency of apo-peptide and their in-situ assembled Zn-peptide complex.

| **Varients** | **Kcat/K_M_ (M^-1^S^-1^)** | |
| --- | --- | --- |
|  | **Peptide** | **Zn-Peptide** |
| **A3** | 0.092 | 1.52 |
| **B1** | 0.12 | 1.58 |
| **B5** | 0.14 | 1.60 |
